# Examining Attitudes Conducive to Technology-Facilitated Child Sexual Exploitation and Abuse: Evidence From a Representative Multi-Country Study

**DOI:** 10.1177/08862605251403621

**Published:** 2026-03-03

**Authors:** Michael Salter, Tyson Whitten, Delanie Woodlock, Carleigh Slater, Ashleigh McFeeters, Mengyao Lu, Georgia Naldrett, Matt Tyler, Deborah Fry

**Affiliations:** 1 University of New South Wales, Sydney, Australia; 2 University of Edinburgh, Scotland; 3 Jesuit Social Services, Melbourne, Australia

**Keywords:** aetiology, child maltreatment, offenders/perpetrators, child maltreatment, sexual abuse, child maltreatment, internet and abuse, technology- or image-based abuse

## Abstract

The development of primary prevention efforts to reduce child sexual abuse before it occurs has been inhibited by a lack of research into the attitudes and beliefs associated with child abuse and maltreatment. This article presents findings from 4,918 men pooled from nationally representative surveys of men in Australia, the United Kingdom and the United States, and presents a latent class analysis of men’s attitudes to technology-facilitated child sexual exploitation and abuse, and its relationship with sexual interest in children and/or sexual offending against children. This study identified and described three latent classes of attitudes towards technology-facilitated child sexual exploitation and abuse, identifying significant and meaningful behavioural and demographic differences between the three groups of men. An important finding of this study is that the shift from “normalisation/blame diffusion” (associated with the belief that technology-facilitated child sexual exploitation and abuse is normal and acceptable behaviour) to “denial of abusiveness: and restrictive stereotypes” (associated with denying that abuse is harmful and desired by the child) was associated with greater odds of acting on sexual interest in children. The finding suggests that the moral or ethical quandary posed by sexual interest in children, and the consensus that child sexual abuse is morally wrong, has an important role to play in the prevention of technology-facilitated child sexual exploitation and abuse. Interventions that seek to reinforce attitudes that child sexual abuse is harmful, and the fault of the perpetrator, may prevent at-risk men from offending. From the perspective of secondary prevention, targeting men who hold normalisation and blame diffusion beliefs may assist agencies in identifying offenders earlier in their offending trajectory. The findings also underscore the critically important role of the media and other sources of cultural influence, including the technology sector and entertainment industry, in reinforcing the moral wrong of child sexual abuse.

## Introduction

Public health approaches to the prevention of interpersonal violence and abuse have targeted social norms and attitudes as part of coordinated efforts to reduce the prevalence of violence (Krug et al., 2002). For instance, efforts to prevent violence against women have included social marketing and educational campaigns that aim to build the social consensus that this abuse is wrong, and to promote bystander intervention, disclosure and help seeking. However, child sexual abuse prevention strategies have generally focused on situational crime prevention as well as educational programmes and campaigns that aim to inform children and caregivers of the risk of abuse, provide them with risk reduction strategies, and emphasising the importance of disclosure and help-seeking in the aftermath of abuse. In relation to technology-facilitated child sexual abuse and exploitation, these prevention strategies are deployed in an uphill battle with social media and other online services that have few child protection obligations under law and have been found to be egregiously unsafe for children (Salter et al. 2023). Other than the deterrence effect of law enforcement, there has been few *primary* prevention efforts that target potential offenders (rather than seeking to inform and upskill children or caregivers) although *secondary* prevention strategies are expanding to target adults who are already offending or on a pathway to offending (Austin & Salter, 2024).

Child sexual abuse prevention scholars have recommended that prevention efforts should identify and target norms and attitudes that legitimise the abuse of children (Quadara et al., 2015; UNICEF, 2020). However, the development of larger-scale social norms intervention into child sexual abuse has been inhibited by a lack of research into the attitudes and beliefs associated with child abuse and maltreatment (Klika et al., 2019). In order to address this gap, this article presents the findings from 4,918 men pooled from nationally representative surveys of men in Australia, the United Kingdom, and the United States. In addition to demographic and internet behaviour questions, the survey asked men whether they had any sexual interest in children or had engaged in any online offending against children, as well as 25 items used to measure men’s attitudes to technology-facilitated child sexual exploitation and abuse. After an overview of research on social norms and attitudes towards child sexual abuse, as well as the study methodology, the article describes and examines latent classes of men’s attitudes to technology-facilitated child sexual exploitation and abuse, and its relationship with sexual interest in children and/or sexual offending against children. The findings show significant differences in the collection of attitudes held by men with (a) no sexual interest or offending behaviours, (b) men with sexual interest in children but no offending behaviours, (c) men who sexually offend but report no sexual interest in children, and (d) men with sexual interest in children who also offend against children. The article closes by exploring the implications of these findings for primary prevention and early intervention.

## Literature Review

Efforts to prevent violence and abuse by changing social norms and attitudes are grounded in public health approaches to behaviour change. Initiatives such as social marketing and education programmes contribute to the transformation of social and cultural norms that legitimise violence, protect perpetrators, and blame victims. In the field of sexual violence prevention, it is important to recognise that programmes with a sole focus on attitude change and awareness raising have proven to be, on their own, insufficient to reduce perpetration (Porat et al., 2024); however, at the same time, prevention approaches focused on structural reforms in the absence of cultural change may trigger backlash or increased risk of violence. To take an example, in an effort to prevent child marriage in India, the age of consent was raised from 16 to 18; however, this reform inadvertently empowered religious and social conservatives to prosecute teenagers having consensual sex (Pitre & Bandewar, 2024). In some regions of India, it is estimated that over half of all prosecutions for statutory rape involve consensual adolescent relationships (Pitre & Bandewar, 2024). Problematic social norms and attitudes are therefore important targets within a comprehensive prevention strategy that addresses a range of determinants or risk factors for child sexual abuse.

Social norms approaches to child sexual abuse prevention are rare, although there is evidence of a link between social norms and attitudes and rates of child sexual abuse. The slow decline over time in rates of adult contact sex offending against children in jurisdictions such as the United States since the 1980s suggests, at the very least, that increased awareness of child sexual abuse and renewed consensus of its wrongfulness may have had a preventative effect (Finkelhor & Jones, 2006). While similar declines have been documented in other countries, rates of child sexual abuse remain nonetheless very high (at 28.5% in Australia, for instance), and declines in adult offending have occurred alongside increased rates of sexually harmful behaviours amongst children (Mathews et al., 2024). It is also unclear the extent to which declines in adult contact offending against children are offset by increased rates of online offending. Nonetheless, social norms and attitudes towards child sexual abuse can be understood as contributing to the overall prevalence of child sexual alongside other risk factors (Quadara et al., 2015; UNICEF, 2020).

This link is particularly evident at the individual level, where attitudes and beliefs that are supportive of child sexual abuse can be an important precursor to offending (Ward & Keenan, 1999). At the individual level, offence-supportive beliefs amongst child sex offenders have often been labelled as “cognitive distortions” (i.e., irrational thoughts and beliefs about the self and the world, see Steel et al., 2020). Cognitive distortions can develop prior to offending as part of a justificatory pathway to perpetration, as well as after offending by way of mitigating feelings of guilt and shame (O’Ciardha & Ward, 2013). These types of rationalisations can been understood as defence mechanisms used by the perpetrator to deflect negative personal and intrapersonal perceptions of their behaviour (Nunes & Jung, 2013). Common cognitive distortions amongst child sex offenders include denial or minimisation of harm to the child victim and blaming the victim for their abuse (Brown, 2020; Steel et al., 2023). Offenders may also reframe abuse as “teaching” or “mentoring” the child victim (Paquette & Cortoni, 2022), or view their abusive behaviours as uncontrollable and a response to other people’s actions (Bartels & Merdian, 2016).

Such beliefs are culturally situated and vary between contexts. For instance, a systematic review of 55 articles on child sexual exploitation (i.e., the exchange of sexual abuse for money or other benefits) identified that, just as forms of child sexual exploitation varied by region, so too did the social norms and attitudes that legitimised this behaviour (Buller et al., 2020). Relevant social norms also vary between “online” and “offline” offending, including the belief that technology-facilitated offending and viewing child sexual abuse material (CSAM) are not “real” forms of abuse, inflicts little or no harm to the victim, and that children depicted in CSAM are sexually sophisticated and consenting to their abuse (Paquette & Cortoni, 2022). Research has also shown that some child sex offenders participate in communities with other offenders, and this communal interaction supports the normalisation of cognitive distortions which may lead to higher rates of perpetration (Salter, 2013; Huikuri, 2023; Shelton et al., 2016).

Such cognitive distortions may also be perpetuated by the media, within social groups, and across communities due to myths surrounding child sexual abuse and victimisation. Scholarship on child sexual abuse myths has studied “incorrect beliefs and stereotyped assumptions about Child Sexual Abuse (CSA), victims, and perpetrators” that circulate collectively (Cromer & Goldsmith, 2010, p. 619). Collings (1997) devised the Child Sexual Abuse Myth Scale to reliably assess adherence to these myths and stereotypes, identifying that they cluster in three key areas: blame diffusion (persons other than the offender are to blame e.g., the child, the non-offending parent or homosexual individuals); denial of abusiveness (portraying abuse as a benign/positive experience; or the child as an equal/consensual partner); and restrictive stereotypes (denial of the reality of most abuse or denial/minimisation of the undesirable consequences of abuse). de Roos et al.’s (2023) study used the Child Sexual Abuse Myth Scale in a survey (and vignette methodology) with undergraduate students and found that endorsement of abuse myths was positively related to racism, sexism, and sexual prejudice. Moreover, belief in child sexual abuse myths was associated with greater likelihood of disbelieving the accuracy of child sexual abuse disclosures. DeMarni et al. (2007) found that female college students disagreed more with abuse myths than male college students did, and female participants believed disclosures more than male participants. These studies suggest that sexism and other forms of prejudice are positively correlated with holding child sexual abuse myths and disbelieving disclosures of child sexual abuse. One study utilising another child sexual abuse myth scale (the CogDIS) found that these myths were significantly more common in child sex offenders diagnosed with paedophilia than in men in the general community (Eberhaut et al., 2023).

The media plays a significant role in the construction of public knowledge and perceptions of abuse, victims/survivors and abusers (Weatherred, 2015). On one hand, the media can contribute to abuse myths that “deny or justify the sexual exploitation of children” (Cromer & Goldsmith, 2010, p. 637). For example, in Collings and Bodill’s (2003, p. 173) work, media depictions of child sexual abuse which used consensual language (e.g., referring to child sexual abuse as an “affair”) were “positively correlated with attributions of causal blame and moral responsibility to the victim.” On the other hand, the media can augment the demonisation of people with paedophilia, whereby simplistic and sensationalised terms such as “‘bad’ (evil), ‘mad’ (mentally disturbed) or ‘sad’ (inadequate)” are used to describe individuals “whose actions are divorced from any social causes” (Wilczynski & Sinclair, 1999, p. 276). To improve media reporting, government and non-government organisations have released guidance for journalists on the content and quality of press coverage of child sexual abuse matters. These guidelines are intended to reduce the prevalence of abuse myths in the mass media, and hence contribute to the prevention of child sexual abuse, as well as to ensure that victims and survivors of child sexual abuse are not unduly impacted by inaccurate or stigmatising coverage (National Children’s Advocacy Centre, 2018; News & Media Research Centre, 2023).

Another key strategy for challenging abuse myths and targeting problematic social norms has been social marketing campaigns. In relation to child sexual abuse, social marketing campaigns have generally focused on increasing knowledge about child sexual abuse, reducing shame and stigma for victims, and encouraging disclosure and bystander intervention (Horsfall et al., 2010). More recently, secondary prevention services such as Stop It Now! have utilised online warning messages targeted at men who may be trying to find CSAM to encourage them to access a helpline or online resources, which in turn aim to promote behavioural and attitude change (Walsh et al., 2023).

Despite evidence of the link between social norms and attitudes, offender beliefs, and offending against children, there is a lack of research into the community-level attitudes and beliefs about child sexual abuse. A key aim of this article is to examine the underlying attitudes that distinguish men based on their sexual interest and sexual offending against children in order to inform prevention and early intervention efforts.

## Methods

### Data

An online survey was conducted examining the prevalence and factors associated with men’s sexual attitudes, interest, and behaviours towards children. Data were drawn from three quota-based samples of men aged 18 years or over comparable to the Australian, U.K., and U.S. male populations in terms of age, residential region, annual household income, and educational attainment. Survey recruitment and administration was conducted by CloudResearch (https://www.cloudresearch.com), an online research panel company with access to an international pool of over 1.5 million participants. The survey was reviewed by a project advisory group which includes representatives from law enforcement, financial intelligence units, government departments, and mental health support services. Surveys were administered from November to December 2022. Ethical approval for this study was provided by the University of New South Wales Human Research Ethics Committee (HC220317).

Initial data were provided by 7,334 respondents (Australia = 2,697; United Kingdom = 2,240; United States = 2,240). Participants were then excluded if they indicated that they were either female at birth, did not identify as male, failed the mid-survey attention check, or reported that they had not answered the questions honestly (*n* = 2,348 removed). Selection bias was reduced by applying population weights to the Australian (*n* = 1,965), U.S. (*n* = 1,502), and U.K. (*n* = 1,519) samples using iterative proportional fitting based on six demographic factors (i.e., race, marital status, employment status, age, annual household income, and educational attainment) sourced from each country’s respective 2021 census. For the current study, data from the three countries were pooled (*n* = 4,918) to increase statistical power.

### Measures

#### Technology-Faciiltated Child Sexual Exploitation and Abuse, and Sexual Interest in Children

Participants were asked nine questions regarding if, during adulthood, they had any sexual interest or engaged in the technology-facilitated sexual exploitation of children. Participants were coded as having sexual interest in children if they answered yes to any of the following questions: (a) I would have sexual contact with a child between 12 and 14 years if no one would find out; (b) I would have sexual contact with a child between 10 and 12 years if no one would find out; (c) I would have sexual contact with a child younger than 10 years if no one would find out; (d) the lowest age I typically find attractive is 15 years or younger; and (e) the highest age I typically find attractive is 15 years or younger.

Participants were coded as having had engaged in the technology-facilitated sexual exploitation of children if they answered yes to any of the following: (a) I knowingly and deliberately view pornographic material containing people below the age of 18; (b) I have flirted or had sexual conversations with a person below the age of 18 online; (c) I have engaged in a sexually explicit webcam interaction with a person below the age of 18; and (d) I have paid for online sexual interactions, images, or videos involving a person below the age of 18. Three non-overlapping categories were created indicating if participants had (a) no sexual interest or technology-facilitated abuse, (b) has sexual interest but no of children, and (c) engaged in online exploitation with or without sexual interest in children.

Participants also responded (0 = *no*; 1 = *yes*) to the following hypothetical scenarios: (a) if given the chance would you watch a webcam sex show of a person under the age of 18 years; (b) if you were sure you were anonymous online, would you watch pornographic material containing people below the age of 18 years, and; (c) would you have sexual contact with a child aged 14 years or under if you were certain that you would not be caught.

#### Demographic Characteristics

Participants reported their sexual orientation (0 = *not heterosexual*; 1 = *heterosexual*), if they had ever had sex with men (0 = *no*; 1 = *yes*), current relationship status (0 = *single, widowed, divorced, or separated*; 1 = *married or living with partner*), employment over the last 3 months (0 = *unemployed*; 1 = *casual, part-time, or full-time employment*), highest educational attainment (0 = *did not obtain a bachelor’s degree*; 1 = *bachelor’s degree or higher*), number of children living in the household (0 = *none*; 1 = *one or more*), if their current work involves contact with children (0 = *does not work with children*; 1 = *works with children*), age (1 = *18–34* *years*; 2 = *35–64* *years*; 3 = *65* *years or older*), total U.S. standardised household income before taxes during the last 12 months (1 = *less than US$25,000 equivalent*; 2 = *between US$25,000–US$99,999*; 3 = *US$100,000 or more*), and residential location (1 = *city*; 2 = *suburb*; 3 = *rural or regional*).

#### Online Pornography Habits

Respondents indicated (0 = *no*; 1 = *yes*) if, while over the age of 18 years, they: (a) purchased online sexual services from another person; (b) knowingly and deliberately viewed pornography involving sex between humans and animals; (c) watched pornography that included sex with violence or force; (d) were ever approached online by an adult offering sexual images, videos, or content; and (e) were ever approached online by a person under 18 years offering sexual images, videos, or content. Participants also reported how often (1 = *never*, 2 = *less than monthly*, 3 = *monthly*, 4 = *weekly*, 5 = *daily*) they accessed sexually explicit websites.

#### Social Media Platform Use

Participants indicated (0 = *no*, 1 = *yes*) if they used any of the following social media platforms: (a) YouTube; (b) Instagram; (c) Facebook; (d) Snapchat; (e) Facebook messenger; (f) TikTok; (g) WhatsApp; (h) Twitter; (i) Discord; (j) Skype; and (k) Viber. Participants also reported how often (1 = *never*, 2 = *less than monthly*, 3 = *monthly*, 4 = *weekly*, 5 = *daily*) they used social media.

#### Attitudes Towards Child Sexual Exploitation

Twenty-five items adapted from the Child Sexual Abuse Myth Scale (Collings, 1997) were used to measure men’s attitudes towards child sexual exploitation. The original scores for each item ranged from *strongly disagree* (1) to *strongly agree* (5). Five items were reverse coded to ensure that higher scores corresponded to greater agreement with attitudes endorsing child sexual exploitation.

Principal components analysis with varimax rotated factor loading and Kaiser normalisation was then conducted to reduce the 25 items from the adapted Child Sexual Abuse Myth Scale into a discernible set of factors. Four components had an eigenvalue greater than 1 and collectively explained 56.9% of the variance (see Table 1). One component only contained two items and was not retained. The three remaining components were designated “denial of abusiveness” (14 items), “normalisation/blame diffusion” (5 items), and “restrictive stereotypes” (4 items), drawing on and partly adapting the three factors identified by Collings (1997). “Denial of abusiveness” refers to beliefs that seek to minimise the harmful nature of technology-facilitated child sexual exploitation and abuse, including by minimising or denying the harm of child sexual abuse, by reframing abuse as positive or beneficial for the child, or by characterising children as equal partners in exploitation. “Normalisation/blame diffusion” refers to beliefs that technology-facilitated child sexual exploitation and abuse is acceptable behaviour, including where potentially mitigating factors are present. “Restrictive stereotypes” refers to stereotypical excuses about people who sexually abuse children online, such as the belief that online abuse may prevent offline abuse, or that technology-facilitated offending may be caused by stress or trauma.

**Table 1.** Principal Components Analysis Varimax Rotated Factor Loading From the Adapted Child Sexual Abuse Myth Scale (*N* = 4,918).

		Component			
#	Item	1	2	3	4
Component 1: Denial of Abusiveness					
1	Sexual images of a person under 18 online where they seem to be happy and enjoying the activity cannot really be described as “abusive.”	**0.62**	0.11	0.09	0.16
2	It is not harmful to look at nude images of someone under 18 if they took the photo of themselves.	**0.71**	0.11	0.14	0.13
3	There is nothing wrong with sex dolls that look like children.	**0.64**	0.20	0.20	0.19
4	Viewing a nude or sexual image of a person under 18 is a victimless crime if the person does not know that the image was taken.	**0.63**	0.04	0.06	0.06
5	I would still be friends with someone who I knew looked at nude or sexual images of people under 18.	**0.69**	0.13	0.25	0.05
6	Boys under 18 are sexually experimental and are not harmed when they interact sexually with an adult online.	**0.75**	0.14	0.16	0.18
7	It’s OK to flirt with people under 18 online if you do not intend to take it further.	**0.75**	0.13	0.18	0.09
8	If a 14- or 15-year-old teenager is on a dating app and contacting adults, they are at least partly responsible if an adult has a sexual interaction with them.	**0.53**	−0.24	0.14	−0.38
9	Online sexual contact with a person under 18 that does not involve actual physical sexual contact or force is unlikely to harm that person psychologically.	**0.66**	0.02	0.14	0.02
10	People under 18 on webcams usually come from poor backgrounds and providing them with money for sexual or nude services is helpful.	**0.62**	0.00	0.20	0.04
11	People under 18 can make their own decisions about how much of their bodies they display on webcam.	**0.69**	0.01	0.18	−0.03
12	People under 18 who offer nude or sexual activity on livestream are exploring their sexuality and should not be censored.	**0.73**	0.16	0.22	0.08
13	I would still be friends with someone who I knew had webcammed or livestreamed sexually with a person under 18.	**0.69**	0.20	0.30	0.06
14	Viewing sexual images or videos of children is bad only because society says it is.	**0.56**	0.20	0.36	0.10
Component 2: Blame diffusion					
15	Drawn, cartoon, or computer-generated sexual imagery of children is not wrong (*reverse coded*).	0.11	**0.73**	0.03	−0.03
16	People under 18 cannot consent to online sexual interactions with adults (*reverse coded*).	0.06	**0.68**	−0.01	−0.17
17	I would not be friends with someone who I knew had sexually interacted online with a person under 18 (*reverse coded*).	0.19	**0.74**	0.01	−0.06
18	It is always wrong to pay to view sexual activity with a child on a webcam, even if the child comes from a poor family and their parents need the money (*reverse coded*).	0.11	**0.80**	−0.05	0.00
19	If someone looks at online sexual images of people under 18 while under the influence of drugs and alcohol, they are still responsible for their actions (*reverse coded*)	0.10	**0.78**	−0.05	0.04
Component 3: Restrictive stereotypes					
20	Sometimes people look at sexual images or videos of children because they are bored with normal adult pornography.	0.44	0.00	**0.76**	−0.01
21	Sometimes people look at sexual images or videos of children because they are very stressed.	0.36	0.09	**0.77**	0.04
22	Some people look at sexual images or videos of children online to prevent themselves from sexually abusing children offline.	0.36	0.01	**0.74**	0.09
23	Some people look at sexual images or videos of children because they were abused when they were children.	0.13	−0.30	**0.61**	0.06
Component 4: Uncategorised					
24	Girls under 18 who share images of themselves nude or in revealing clothing are not to be blamed if an adult responds to them in a sexual way.	0.23	−0.13	0.06	**0.79**
25	People under 18 who act in sexual ways online are not to blame if an adult responds to them in a sexual way.	0.22	−0.16	0.11	**0.79**
Rotation Sums of Squared Loadings	Eigenvalue	6.77	3.21	2.67	1.59
	Percentage of variance	27.09%	12.84%	10.70%	6.34%

*Note.* KMO = 0.943, Bartlett’s test of sphericity *x*^2^ = 54,946.67 (*df* = 300), *p* < .001. Bold values indicate retatined items for each component.

There was good internal and cross-country consistency as evidenced from the Cronbach’s alpha (α) and mean inter-item correlations (IIC) for the items comprising “denial of abusiveness” (Australia α = .92, IIC = .46; United Kingdom α = .91, IIC = .42; United States α = .93, IIC = .49), “normalisation/blame diffusion” (Australia α = .87, IIC = .57; United Kingdom α = .77, IIC = .41; United States α = .81, IIC = .47), and “restrictive stereotypes” (Australia α = .81, IIC = .51; United Kingdom α = .78, IIC = .47; United States α = .80, IIC = .50).

### Analytic Strategy

Distinct patterns of attitudes towards child sexual exploitation were identified using Latent Class Analysis (LCA); a statistical method used to identify discrete classes of individuals that reflect their underlying traits based on their responses to a series of categorical variables (Lanza et al., 2007). Individuals are clustered into latent classes based on the posterior probabilities of class membership. Each latent class also encompasses a conditional probability indicating the likelihood that individuals from that class endorse the relevant child sexual exploitation attitude. The latent class model makes no assumptions about the distribution of categorical indicators other than that they are independent within each latent class (otherwise known as local independence).

In preparation for LCA, scores from the adapted Child Sexual Abuse Myth Scale were dichotomised to indicate agreement (2 = *agree and strongly agree*) or non-agreement (1 = *strongly disagree, disagree, and neither agree or disagree*). The LCA was conducted using the PROC LCA procedure for SAS (v. 9.4). No cases had missing data for the grouping variables. A two, three, four, five, and six latent class solution were considered. Model fit was evaluated based on log-likelihood and entropy, as well as the Akaike’s Information Criterion (AIC) and the Bayesian Information Criterion (BIC), both of which are penalised log-likelihood model information statistics. These statistics are used to compare model fit among competing models using the same data, with smaller values relative to the reduction in the degrees of freedom indicating better model fit and parsimony (Lanza et al., 2007). Models producing class sizes greater than 5% and average posterior probabilities greater than 90% were preferred.

Descriptive statistics were provided for all participants included in the study and separately for each latent class. Weighted logistic regression analyses adjusted for country were conducted to identify the characteristics that differentiate each latent class. Odds ratios (ORs) and 95% confidence intervals (95% CIs) were reported as measures of effect size and precision of the association between the covariates and outcome variables. Results were calculated using survey weights, with robust standard errors (Lumley, 2004). All assumptions underpinning logistic regression analysis were met (Tabachnick & Fidell, 2013). Analyses are exploratory and were not adjusted for multiple comparisons. Analyses were conducted in IBM SPSS version 29 (IBM Corp, 2022).

## Results

**Figure 1.**
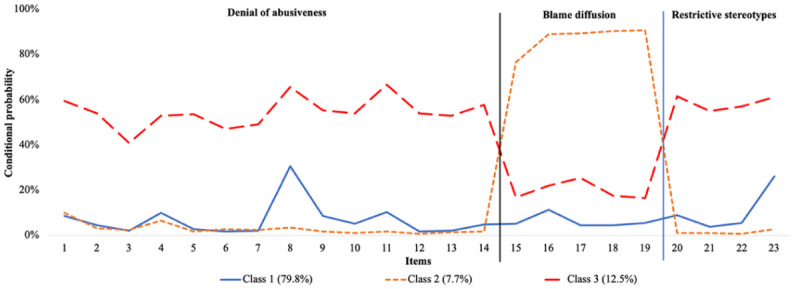
Three model solution for latent classes of child sexual exploitation attitudes (*n* = 4,918).

Test statistics from the LCA indicated that the three-class solution was the optimal model (see Table 2), revealing three clearly distinguishable classes (see Figure 1). Most participants were assigned to Class 1 (*n* = 3,926, 79.8%), which was characterised by a low probability of endorsing any attitudes conducive to child sexual exploitation. Class 2 was the smallest group (*n* = 380, 7.7%), and encompassed participants who had the lowest probability of endorsing items relating to “denial of abusiveness” and “restrictive stereotypes,” but the highest probability of agreeing with attitudes relating to “normalisation/blame diffusion.” Finally, one in eight people was designated Class 3 (*n* = 612, 12.5%), which had a moderately high probability of endorsing items corresponding to “denial of abusiveness” and “restrictive stereotypes,” and a moderately low probability of endorsing items relating to “normalisation/blame diffusion.”

**Table 2.** Latent Class Analysis Model Fit Statistics (*N* = 4,918).

#	Latent class proportions (average posterior probabilities)						AIC	ΔAIC	BIC	ΔBIC	Log-Likelihood	Entropy
1	100% (100%)						89,678.7	—	89,828.3	—	−44,816.4	—
2	12.4% (96.2%)	87.6% (99.2%)					76,763.0	12,915.7	77,068.6	12,759.7	−38,334.5	.970
**3**	**79.8% (98.7%)**	**7.7% (97.4%)**	**12.5% (96.8%)**				**71,830.1**	**4,932.9**	**72,291.8**	**4,776.8**	**−35,844.0**	**.956**
4	17.1% (91.0%)	7.4% (97.4%)	71.4% (96.7%)	4.0% (96.5%)			69,878.9	1,951.2	70,496.6	1,795.2	−34,844.4	.889
5	68.5% (94.4%)	6.1% (78.2%)	3.8% (96.0%)	7.4% (97.1%)	14.1% (89.1%)		69,355.9	523.0	70,129.7	366.9	−34,558.9	.835
6	7.0% (89.7%)	63.2% (92.5%)	6.2% (80.2%)	2.3% (96.6%)	14.4% (79.6%)	7.0% (96.9%)	68,919.0	436.8	69,848.9	280.8	−34,316.5	.731

Bold values correspond to the retained solution.

Table 3 presents the weighted prevalence of each latent class regarding sexual interest and online sexual exploitation of children, demographic characteristics, online pornography viewership, and social media use. A greater proportion of men from the United Kingdom (84.1%) than Australia (79.9%) and United States (75.4%) were designated Class 1 (*x*^2^(2) = 35.51, *p* < .001). A greater proportion of men from Australia (10.0%) than the United Kingdom (5.4%) and United States (7.1%) were in Class 2 (*x*^2^(2) = 26.75, *p* < .001), whereas more men from the United States (17.6%) than Australia (10.1%) and the United Kingdom (10.5%) were in Class 3 (*x*^2^(2) = 51.12, *p* < .001).

**Table 3.** Descriptive Statistics (Column Proportions).

Measures	Class 1 (*n* = 3,926), %	Class 2 (*n* = 380), %	Class 3 (*n* = 612), %
Technology-facilitated child sexual exploitation and abuse (TF-CSEA) and sexual interest			
No interest or TF-CSEA	92.9 [91.9, 93.8]	70.9 [65.2, 76.0]	47.7 [43.1, 52.3]
Interest only	2.6 [2.1, 3.3]	21.5 [17.0, 26.9]	18.6 [15.2, 22.6]
TF-CSEA with/without interest	4.5 [3.8, 5.4]	7.6 [5.2, 11.0]	33.8 [29.7, 38.1]
Would watch webcam	3.2 [2.6, 4.1]	2.6 [1.4, 4.6]	23.3 [19.6, 27.3]
Would watch pornography	2.8 [2.2, 3.5]	7.2 [4.7, 10.8]	21.2 [17.8, 25.0]
Would have sexual contact	1.4 [1.0, 1.9]	21.5 [17.1, 26.7]	36.5 [32.2, 41.0]
Demographic characteristics			
Heterosexual	92.5 [91.5, 93.4]	96.7 [93.7, 98.3]	93.3 [90.7, 95.3]
Had sex with men	13.3 [12.1, 14.6]	23.3 [18.6, 28.8]	31.9 [27.8, 36.4]
Married or living with partner	58.6 [56.8, 60.5]	68.3 [62.4, 73.7]	63.5 [58.9, 67.9]
Employed	61.9 [60.1, 63.7]	84.4 [79.2, 88.5]	77.8 [73.4, 81.6]
Bachelor’s degree or higher	35.2 [33.5, 37.0]	47.5 [41.7, 53.4]	44.8 [40.3, 49.4]
Child in household	28.0 [26.4, 29.7]	56.0 [50.1, 61.8]	47.6 [43.0, 52.2]
Works with children	12.5 [11.4, 13.7]	22.5 [18.3, 27.3]	35.1 [30.9, 39.7]
Residential location			
City	27.5 [25.8, 29.2]	65.0 [59.0, 70.5]	55.0 [50.3, 59.5]
Suburbs	47.4 [45.6, 49.3]	24.3 [19.4, 30.0]	31.5 [27.3, 36.1]
Regional or rural	25.1 [23.6, 26.7]	10.7 [7.7, 14.8]	13.5 [10.5, 17.0]
Age			
18–34 years	27.2 [25.6, 28.9]	47.7 [41.8, 53.6]	41.8 [37.4, 46.4]
35–64 years	50.0 [48.1, 51.8]	46.0 [40.2, 52.0]	42.4 [37.9, 47.1]
65 years and older	22.9 [21.3, 24.5]	6.3 [4.1, 9.6]	15.7 [12.9, 19.1]
Household income			
Less than US$25,000	22.7 [21.1, 24.4]	18.2 [13.7, 23.7]	23.1 [19.0, 27.8]
US$25,000–US$99,999	57.0 [55.1, 58.8]	40.1 [34.5, 45.9]	45.9 [41.4, 50.5]
US$100,000 or more	20.3 [18.9, 21.9]	41.8 [36.1, 47.7]	30.9 [27.0, 35.2]
Country			
Australia	39.5 [37.6, 41.3]	51.2 [45.3, 57.1]	31.9 [27.7, 36.4]
United Kingdom	32.3 [30.6, 34.0]	21.4 [16.9, 26.6]	25.8 [22.0, 30.1]
United States	28.3 [26.7, 29.9]	27.4 [22.6, 32.9]	42.2 [37.8, 46.8]
Online pornography viewership			
Mean pornography frequency	2.38 [2.32, 2.43]	2.90 [2.72, 3.08]	3.35 [3.22, 3.49]
Purchased sexual service	6.2 [5.3, 7.1]	13.8 [10.4, 18.1]	36.8 [32.6, 41.3]
Watches violent or rough porn	9.6 [8.6, 10.8]	5.0 [3.1, 7.9]	24.4 [20.6, 28.6]
Watches bestiality	3.0 [2.4, 3.7]	2.9 [1.6, 5.1]	20.2 [16.7, 24.3]
Approached by adult	19.6 [18.1, 21.1]	8.9 [6.4, 12.2]	37.0 [32.8, 41.4]
Approached by child	4.7 [3.9, 5.6]	6.2 [4.1, 9.5]	24.8 [21.1, 28.9]
Social media use			
Mean social media use frequency	4.17 [4.12, 4.23]	4.26 [4.12, 4.49]	4.32 [4.22, 4.42]
YouTube	72.9 [71.3, 74.5]	69.5 [63.3, 75.0]	83.7 [80.1, 86.7]
Instagram	40.5 [38.7, 42.3]	51.1 [45.2, 57.0]	61.4 [56.7, 65.9]
Facebook	68.5 [66.8, 70.2]	67.4 [61.4, 72.8]	73.8 [69.7, 77.5]
Snapchat	23.3 [21.8, 24.9]	34.2 [28.8, 40.0]	44.6 [40.1, 49.2]
Facebook messenger	45.7 [43.9, 47.5]	41.0 [35.3, 47.0]	52.7 [48.1, 57.3]
TikTok	27.3 [25.7, 29.0]	43.1 [37.3, 49.2]	46.5 [41.9, 51.1]
WhatsApp	38.4 [36.6, 40.2]	40.4 [34.7, 46.4]	58.1 [53.5, 62.6]
Twitter	30.5 [28.8, 32.2]	30.4 [25.3, 36.1]	49.3 [44.8, 53.9]
Discord	22.4 [10.3, 12.6]	11.6 [8.5, 15.6]	22.6 [19.0, 26.7]
Skype	10.0 [8.9, 11.2]	13.0 [10.0, 16.6]	25.6 [21.9, 29.8]
Viber	2.9 [2.3, 3.6]	4.0 [2.0, 7.6]	9.5 [6.9, 12.8]

Class 1 had the lowest number of men who had sexual interest in children (2.6%), engaged in technology-facilitated sexual exploitation and abuse of children (4.5%), would watch a webcam sex show (3.2%) or CSAM (2.8%), or would have sexual contact with a child under 15 years (1.4%). By comparison, around one-in-five men in Class 2 had sexual interest in children (21.5%) and would have sexual contact with a child (21.5%). Around one-in-five men in Class 3 had sexual interest in children (18.6%) and would watch a sexually explicit webcam (23.3%) or pornography (21.2%) of children, while one-in-three had engaged in technology-facilitated child sexual exploitation and abuse (33.8%) and would have sexual contact with a child under 15 years (36.5%).

The weighted odds of latent class membership, adjusted for country, is presented in Table 4. Relative to Class 1, the odds of having sexual interest in children were 10.47 times higher for Class 2 and 13.88 times higher for Class 3. Classes 2 and 3 were also 2.18 and 14.18 times more likely to have engaged in technology-facilitated child sexual exploitation and abuse, 2.78 and 9.00 times more likely to watch child exploitation material if offered, and 19.20 and 39.29 times more likely to have sexual contact with a child under 15 years, respectively. Furthermore, those in Class 3 compared to Class 2 were 6.25 times more likely to engage in technology-facilitated child sexual exploitation and abuse, 11.38 times more likely to watch a sexually explicit webcam of a child, 2.98 times more likely to watch child exploitation material, and 2.09 times more likely to have sexual contact with a child under 15 years.

**Table 4.** Weighted Multinomial and Logistic Regression (OR, 95% CI), Adjusted for Country.

Measures	Class 1 (ref) vs.		Class 2 (ref) vs.
	Class 2	Class 3	Class 3
TF-CSEA and sexual interest			
No interest or TF-CSEA	1.00 (reference)	1.00 (reference)	1.00 (reference)
Interest only	10.47 [7.22, 15.16]	13.88 [9.69, 19.89]	1.39 [0.93, 2.06]
TF-CSEA with/without interest	2.18 [1.38, 3.44]	14.18 [10.79, 18.64]	6.25 [3.92, 9.95]
Would watch webcam	0.80 [0.41, 1.54]	9.35 [6.79, 12.88]	11.38 [5.88, 22.03]
Would watch pornography	2.78 [1.67, 4.63]	9.00 [6.56, 12.34]	2.98 [1.81, 4.92]
Would have sexual contact	19.20 [12.69, 29.06]	39.29 [27.37, 56.52]	2.09 [1.49, 2.92]
Demographic characteristics			
Heterosexual	2.39 [1.20, 4.75]	1.12 [0.76, 1.66]	0.51 [0.23, 1.11]
Had sex with men	2.02 [1.48, 2.74]	3.05 [2.43, 3.84]	1.48 [1.04, 2.10]
Married or living with partner	1.56 [1.18, 2.05]	1.24 [1.00, 1.53]	0.82 [0.58, 1.15]
Employed	3.27 [2.28, 4.69]	2.20 [1.70, 2.85]	0.61 [0.38, 0.96]
Bachelor’s degree or higher	1.69 [1.32, 2.17]	1.59 [1.30, 1.95]	0.92 [0.68, 1.25]
Child in household	3.23 [2.50, 4.17]	2.33 [1.90, 2.85]	0.66 [0.48, 0.90]
Works with children	2.01 [1.52, 2.67]	3.82 [3.06, 4.77]	1.71 [1.23, 2.37]
Residential location			
City	5.26 [3.56, 7.77]	3.66 [2.71, 4.93]	0.70 [0.43, 1.14]
Suburbs	1.07 [0.69, 1.67]	1.26 [0.91, 1.74]	1.15 [0.65, 2.02]
Regional or rural	1.00 (reference)	1.00 (reference)	1.00 (reference)
Age			
18–34 years	6.21 [3.84, 10.03]	2.24 [1.69, 2.98]	0.27 [0.15, 0.50]
35–64 years	3.33 [2.06, 5.37]	1.22 [0.92, 1.61]	0.28 [0.15, 0.53]
65 years and older	1.00 (reference)	1.00 (reference)	1.00 (reference)
Household income			
Less than US$25,000	1.05 [0.72, 1.52]	1.31 [1.00, 1.72]	1.25 [0.78, 2.00]
US$25,000–US$99,999	1.00 (reference)	1.00 (reference)	1.00 (reference)
US$100,000 or more	2.76 [2.11, 3.63]	1.74 [1.40, 2.16]	0.69 [0.49, 0.96]
Online pornography viewership			
Pornography use frequency	1.26 [1.16, 1.37]	1.55 [1.44, 1.66]	1.23 [1.10, 1.37]
Purchased sexual service	2.63 [1.83, 3.77]	8.50 [6.65, 10.87]	3.17 [2.17, 4.65]
Watches violent or rough porn	0.47 [0.28, 0.78]	3.13 [2.43, 4.04]	6.17 [3.53, 10.77]
Watches bestiality	0.96 [0.51, 1.81]	7.80 [5.64, 10.78]	8.19 [4.27, 15.70]
Approached by adult	0.39 [0.27, 0.56]	2.33 [1.89, 2.88]	5.59 [3.70, 8.45]
Approached by child	1.33 [0.82, 2.16]	6.42 [4.85, 8.51]	4.73 [2.87, 7.81]
Social media use			
Social media use frequency	1.04 [0.96, 1.13]	1.09 [1.02, 1.17]	1.05 [0.91, 1.21]
YouTube	0.81 [0.61, 1.09]	1.95 [1.51, 2.51]	2.18 [1.52, 3.13]
Instagram	1.52 [1.18, 1.95]	2.31 [1.88, 2.85]	1.38 [1.01, 1.89]
Facebook	0.92 [0.70, 1.21]	1.27 [1.02, 1.58]	1.38 [0.99, 1.93]
Snapchat	1.69 [1.29, 2.20]	2.58 [2.10, 3.18]	1.45 [1.06, 1.99]
Facebook messenger	0.80 [0.62, 1.03]	1.33 [1.09, 1.62]	1.50 [1.10, 2.05]
TikTok	1.99 [1.53, 2.57]	2.26 [1.84, 2.77]	1.05 [0.77, 1.45]
WhatsApp	1.23 [0.94, 1.61]	2.90 [2.31, 3.64]	2.05 [1.50, 2.80]
Twitter	1.02 [0.78, 1.33]	2.24 [1.83, 2.74]	2.09 [1.51, 2.89]
Discord	0.96 [0.66, 1.38]	2.39 [1.85, 3.07]	2.42 [1.58, 3.69]
Skype	1.31 [0.96, 1.80]	3.14 [2.47, 4.00]	2.35 [1.64, 3.37]
Viber	1.29 [0.62, 2.70]	3.80 [2.52, 5.73]	2.51 [1.17, 5.39]

Regarding demographic characteristics, men from Classes 2 and 3 were significantly more likely than those from Class 1 to have ever had sex with men (OR = 2.02–3.05), be married or living with partner (OR = 1.56–1.24), employed (OR = 3.27–2.20), have a bachelor’s degree or higher (OR = 1.69–1.59), have a child in the household (OR = 3.23–2.33), work with children (OR = 2.01–3.82), live in the city (OR = 5.26–3.66), be aged 18 to 34 years (OR = 6.21–2.24), and have an annual household income of US$100,000 or more (OR = 2.76–1.74). By contrast, men in Class 3 were significantly more likely than those from Class 2 to have ever had sex with men (OR = 1.48), not be employed (OR = 0.61), not have a child in the household (OR = 0.66), work with children (OR = 1.71), be aged 65 years or older (OR = 3.70), and have an annual household income of less than US$100,000 (OR = 0.69).

In terms of online pornography viewership, men from Classes 2 and 3 were significantly more likely than those from Class 1 to watch pornography more frequently (OR = 1.26–1.55) and purchase sexual services online (OR = 2.63–8.50). However, men from Class 2 were significantly less likely than men from Class 1 to intentionally watch violent or rough porn (OR = 0.47) or ever be approached by an adult selling sexual services online (OR = 0.39). By contrast, men from Class 3 compared to men from Class 1 were 3.13 times more likely to watch violent or rough porn, 7.80 times more likely to watch bestiality, 2.33 times more likely to be approached by an adult selling sexual services online, and 6.42 times more likely to have been approached by a child selling sexual services online. Similar patterns of association emerge when comparing men from Classes 2 and 3.

Regarding social media platform use, men from Class 3 were significantly more likely to use social media more frequently (OR = 1.09) and were more likely to engage in all social media platforms than men from Class 1. Men from Class 2 were significantly more likely than men from Class 1 to use Instagram (OR = 1.52), Snapchat (OR = 1.69), and TikTok (OR = 1.99). Men from Class 3 were significantly more likely to use all social media platforms except for Facebook and TikTok than men from Class 2.

## Discussion

This is the first study to explore attitudes around child sexual exploitation among representative community samples of men in three countries. This study identified and described three latent classes of attitudes towards child sexual exploitation, identifying significant and meaningful behavioural and demographic differences between the three groups of men. Class 1 had the lowest probability of endorsing any attitudes conducive of technology-facilitated child sex offending, and the lowest rates of sexual interest or offending against children. This was the largest group in the survey, although considerably larger in the United Kingdom and Australia compared to the United States.

Men in Class 2 had the highest probability of endorsing “normalisation/blame diffusion” attitudes, a set of beliefs that ascribe responsibility for abuse to people or circumstances other than the offender. Normalisation and blame diffusion was clearly associated with significant risk to children. This group of men were over 10 times more likely to have sexual interest in children compared to Class 1. One in five men in Class 2 had sexual interest in children, and one in five had sexually offended against children. Compared to Class 1, this group indicated that they were almost 20 times more likely to have sexual contact with a child under 15 if nobody would find out. They were also more likely to watch pornography frequently and purchase sexual services online.

Compared to Class 1, men in Class 2 had a distinctly privileged demographic profile and were more likely to have access to children at home and work. They were over three times more likely to be employed, almost twice as likely to have a bachelor’s degree, over three times more likely to have a child in the house, twice as likely to work with children, over five times more likely to live in the city, over six times more likely to be young (aged 18–34), and almost three times more likely to have an annual household income over US$100 000. The reason why this group of men were, on average, more successful than Class 1 men in terms of educational attainment and socioeconomic status is beyond the scope of this article but deserves further inquiry.

Men in Class 3 represented a higher risk to children than Class 2. Compared to Class 2, men in Class 3 were over six times more likely to have engaged in technology-facilitated child sexual exploitation and abuse and over two times more likely to indicate that they would have sexual contact with a child under 15 years if they knew no one would find out. They displayed a significantly different attitudinal posture to Class 2. This group had a moderately high probability of endorsing items corresponding to “denial of abusiveness” (beliefs that minimise the abusive or harmful nature of child sexual abuse) and “restrictive stereotypes” (denial of the facts about child sexual abuse), and a moderately low probability of endorsing items relating to “normalisation/blame diffusion.” This group was 17.6% of men in the United States, and one in 10 men in Australia and the United Kingdom.

Compared to Class 1, Class 3 had a very similar demographic profile to Class 2. While these two classes were more likely to report sex with men than Class 1, however these groups were not more likely to identify with a non-heterosexual orientation. It should be noted that some men who sexually abuse children can engage in “sexually indiscriminate” behaviour including the abuse of children and sexual contact with women and men (Miller, 2013). Compared to Class 2, men in Class 3 had a somewhat diminished demographic profile: less likely to be employed, less likely to have a child in the house, less likely to earn over US$100,000, and over three times more likely to be over 65. Nonetheless, they were 1.7 times more likely than men in Class 2 to work with children, and their online sexual behaviour was considerably more intensive and illegal. Compared to Class 2, men in Class 3 were over three times more likely to watch violent or rough porn, almost eight times more likely to watch bestiality, over two times more likely to be approached by an adult selling sexual services online, and over six times more likely to have been approached by a child selling sexual services online. They were significantly more likely to use all social media platforms except for Facebook and TikTok than men from Class 2.

Collectively, these findings have identified two classes of men who pose a risk to children according to their attitudes towards technology-faciltated child sexual exploitation and abuse. Men in Class 2 had a high level of sexual interest in children, and one in five had engaged in technology-facilitated sexual offending against children. Their attitudes towards technology-facilitated offending were characterised by normalisation and blame diffusion but, comparatively, low restrictive stereotypes and denial of abuse which may suggest that they are aware of the harms of abuse but misallocate blame externally. This may be indicative of an ongoing moral or ethical struggle around the implications of their sexual interest and/or behaviour towards children. Their endorsement of normalisation/blame diffusion suggests that they believed there was some blame – that is, responsibility – to diffuse in relation to their wish to sexually abuse children. This was a comparatively young and affluent group of men.

Compared to Class 2, men in Class 3 were engaged in much higher levels of technology-facilitated sexual offending against children, as well as higher levels of online deviancy, including viewing pornography featuring violence and sex activity with animals. These rates of technology-facilitated offending and other deviancy were associated with a specific attitudinal posture that differentiated them from Class 2: namely, this group did not believe, on the whole, that child sexual abuse was wrong or harmful. This group did not evince the normalisation and blame diffusion of Class 2, potentially because they do not believe that their behaviour is wrong; hence, there is no blame to diffuse. Instead, this group is intensively engaged in technology-facilitated offending against children, which is justified by their attitudes and beliefs.

The demographic findings of this study are of interest, namely that men in Classes 2 and 3 were better educated than men in Class 1, dispelling any myths that their offence-supportive attitudes and beliefs result from insufficient education. On the contrary, it may be that more education may supply child sex offenders with a myriad of sources to rationalise and legitimise the sexual abuse of children. An important finding of this study is that the shift from normalisation and blame diffusion, associated with wanting to sexually abuse children, to denial of abusiveness and restrictive stereotypes is associated with greater odds of acting on that sexual interest. Men characterised by normalisation/blame diffusion were, on average, younger than men characterised by denial of abusiveness and restrictive stereotypes, which gives rise to two possibilities. The first is that the study has identified a shift over time in attitudinal posture as men with a sexual interest in children move down an offending pathway. However, this is a cross-sectional rather than longitudinal study; hence, any such inference should be highly qualified. It is also relevant to note the somewhat diminished demographic profile of men in Class 3 compared to men in Class 2, alongside the higher level of sexual preoccupation evident in men in Class 3, which suggests that they may be a particular cohort of sexually preoccupied and less socially successful group of men. A second possibility is that the attitudinal differences identified in the study between Classes 2 and 3 is generational, and linked to attitudinal differences between different age cohorts.

## Conclusion

This is the first study to explore attitudes towards child sexual exploitation and abuse by representative community samples of men in Australia, the United Kingdom, and the United States. The finding suggests that the moral or ethical quandary posed by the desire to sexually abuse children has an important role to play in prevention and that interventions that seek to reinforce attitudes that child sexual abuse is harmful, and the fault of the perpetrator, may prevent at-risk men from offending. From the perspective of secondary prevention, targeting men who hold normalisation/blame diffusion beliefs may assist agencies in identifying offenders earlier in their offending trajectory. The findings also underscore the critically important role of the media and other sources of cultural influence, including the technology sector and entertainment industry, in reinforcing the moral wrong of child sexual abuse and the responsibility of offenders rather than victims. Recent content analysis showing that the most common form of sexual violence depicted in adult pornography is incest also raises questions about the role of adult content in undermining social consensus against child sexual abuse (Vera-Gray et al., 2021) and the place of content and media regulation by governments in the prevention of child sexual abuse. It is also notable that pro-paedophile social movements have become increasingly visible on social media with a specific agenda around the normalisation of sexual interest in children (Farmer et al., 2024). Based on the findings of this study, it is very concerning that these groups are promoting beliefs that have an empirical association with the sexual abuse of children.

The study has a number of limitations. Firstly, the survey is not based on a probability sample but rather recruited through an online survey panel company for practical and cost-effective reasons, which may impact on the generalisability of findings. Secondly, the findings are based on self-report data which may introduce social desirability bias due to the sensitive nature of the questions. Third, by defining a child as any person under the age of 18 for the purposes of measuring technology-facilitated offending, the study may have inadvertently categorised some lawful activity as unlawful. Children were defined as any person under the age of 18 because this is a multi-jurisdictional survey and the age of consent for sexual activity (online and offline) varies within and between the three countries, and has varied over time due to changes to the law. While it was not feasible for the survey to undertake a granular analysis of the exact criminal status of all reported sexual acts, future iterations of the survey and perpetration prevalence research should consider whether and how to address these jurisdictional variations.
